# Making Newborn Screening Work in Low- and Middle-Income Countries: From Global Commitment to Sustainable Action—The WHO Framework

**DOI:** 10.3390/ijns12040076

**Published:** 2026-09-24

**Authors:** Bradford L. Therrell, Carmencita D. Padilla

**Affiliations:** 1 Department of Pediatrics, Long Medical School, University of Texas at San Antonio, San Antonio, TX 78229, USA; 2 National Newborn Screening and Global Resource Center, Austin, TX 78759, USA; 3 Department of Pediatrics, College of Medicine, University of the Philippines Manila, Manila 1000, Philippines; cdpadilla@up.edu.ph; 4 Newborn Screening Reference Center, National Institutes of Health, University of the Philippines Manila, Manila 1000, Philippines

**Keywords:** newborn screening, World Health Organization (WHO), World Health Assembly (WHA), low- and middle-income countries (LMIC), public health

## Abstract

Newborn screening (NBS) is an established public health intervention capable of preventing death, disability, and lifelong disease through early identification and treatment of affected newborns. However, equitable access to NBS remains limited, particularly in low- and middle-income countries (LMICs), where most serious birth defects occur. The 2024 World Health Assembly resolution WHA77.5 and the newly published World Health Organization (WHO) newborn screening framework provide an important global mandate and practical roadmap for expanding NBS, diagnosis, treatment, and long-term care. The framework reviewed by De Costa et al. emphasizes that NBS is a continuum of care encompassing screening, diagnostic confirmation, treatment, follow-up, rehabilitation, developmental services, and family support. Successful implementation requires quality assurance across the entire pathway, including specimen collection, laboratory testing, reporting, tracking, diagnostic services, and treatment, with external proficiency testing where appropriate. Programs should begin with condition(s) for which disease burden, effective treatment, screening feasibility, and health system capacity are aligned, and should expand progressively as capacity develops. Sustainable financing, trained personnel, information systems, referral networks, and national government stewardship are essential. Rather than replicating high-income-country models, LMICs should develop locally appropriate, scalable programs based on quality, equity, effectiveness, and continuity of care. The challenge now is to translate the WHO framework into sustainable national action.

As neonatal mortality from infectious diseases, malnutrition, and vaccine-preventable illnesses declines, congenital disorders (birth defects) account for an increasing share of neonatal and under-five mortality, and lifelong disability. Effective newborn screening (NBS)—laboratory screening, physical examinations, or clinical tests—is a vital public health intervention capable of preventing severe disability and death through early condition detection and treatment [1].

The global landscape of NBS is changing [2,3,4,5]. For decades, the principal challenge has been to demonstrate that early identification of newborns with treatable conditions can prevent death, disability, and lifelong disease. Today, the challenge is different: how can the benefits of newborn screening be made available to every newborn, including those born in low- and middle-income countries (LMICs)?

This question has acquired new importance following the 2024 World Health Assembly resolution WHA77.5 [6], which called on Member States to consider implementing universal newborn screening (NBS), including comprehensive birth-defect screening, together with diagnosis, management, and long-term care. The WHO framework [7] developed in an extensive WHO Consultation described by De Costa and colleagues in this issue [8] provides an important response to that mandate. It moves the discussion beyond whether LMICs should undertake NBS to the more practical question of how screening can be introduced, strengthened, and sustained within existing LMIC health systems.

As De Costa et al. note, the scale of the problem is substantial. An estimated 3–6% of babies are born with serious birth defects, which represents approximately 8 million newborns annually, with more than 90% of serious birth defects occurring in LMICs. Of the estimated 400,000–500,000 annual childhood deaths associated with birth defects, approximately 95% occur in LMICs. These figures make inequitable access to early identification and treatment not simply a technical problem, but an important global health and health-equity issue.

**Screening Is a System, Not a Test:** Perhaps the most important message in the WHO framework is the renewed emphasis that newborn screening is more than a laboratory test. Screening is the entry point into a continuum of care that includes screening, diagnosis, treatment, rehabilitation, long-term follow-up, and support for families.

This distinction is particularly important when considering newborn screening implementation in LMICs. A high-quality screening test has little public-health value if a newborn with a positive result cannot obtain timely diagnostic confirmation or appropriate treatment. Conversely, a country does not necessarily need to establish an immediately comprehensive program for dozens of conditions before it begins screening. The WHO framework appropriately emphasizes starting with one or a few conditions for which disease burden, effective treatment, screening feasibility, and health system capacity are aligned.

**Quality Must Be Included from the Beginning:** The WHO framework recognizes the importance of quality systems throughout the screening pathway. Quality assurance should be regarded as core program infrastructure and not as an optional component to be added after program implementation.

High quality must exist for all of the pertinent screening components: specimen collection, transport and testing, result reporting, patient tracking/recall, diagnostic confirmation, disease management/treatment, and patient compliance. When laboratories are involved, their implementation must include internal quality control, appropriate reference materials, standardized procedures, quality indicators, external proficiency testing, and mechanisms for corrective action.

This is noted as especially important for LMIC programs establishing or strengthening regional and national screening laboratories. Where individual countries cannot immediately support all necessary specialized laboratory functions, use of regional laboratory networks and shared quality-assurance systems may provide an effective pathway to high-quality testing.

**The Screening Pathway Includes Many Components:** The history of NBS has emphasized analytical technologies—from bacterial inhibition assays and immunoassays to high-performance liquid chromatography, tandem mass spectrometry, molecular analyses, and, increasingly, genomics. However, technological capability alone does not result in an effective NBS program.

Similar to Wilson and Younger criteria [9], the WHO framework suggests that before screening for a disorder, fundamental questions should be addressed: Can the condition be reliably detected? Can a positive result be confirmed rapidly? Is effective treatment available? Can families reach appropriate care? Is long-term follow-up possible? Can outcomes be measured?

The WHO framework appropriately stresses that referral pathways and access to diagnosis and treatment should be established before population-wide NBS is undertaken. This principle is particularly relevant as NBS panels expand. The temptation to equate technological capability with readiness must be resisted. The ability to detect a disorder is not equivalent to the ability to provide beneficial NBS.

**Sustainability Requires National Ownership:** De Costa et al. note the emphasis on sustainable financing and government stewardship as an essential component of the WHO framework. NBS programs that depend indefinitely on short-term research grants, charitable contributions, or external donors remain vulnerable to changes in priorities and funding. This often results in erratic screening efforts that disappear with the funding.

The experiences in the Philippine bloodspot screening program [10] were noted as one example of how NBS can evolve from a program supported initially through fees and external assistance to a national government-supported public health program that includes pertinent integrated services. From a pilot project in a small number of hospitals assessing the incidence of a few disorders, the program expanded substantially over the years to include a much broader screening panel and thousands of participating birthing facilities. Other screening experiences discussed included examples of programs for cardiac, hearing, sickle cell disease, and clubfoot screening.

Such experiences demonstrate that sustainability is not achieved simply by securing an initial source of funding. It requires policy commitment, financing mechanisms, trained personnel, laboratory infrastructure, procurement systems, referral networks, information systems, and institutional responsibility.

**Data Should Follow the Newborn:** The WHO framework highlights examples of developing data systems and continuity-of-care mechanisms for NBS in several countries, including India, Uganda, and the Philippines [7]. It emphasizes the fact that effective NBS programs need information systems capable of following the newborn throughout the screening pathway. Programs must document whether a newborn was screened, whether the result was positive, whether diagnostic confirmation occurred, whether treatment/management began, the level of compliance with treatment/management, and what ultimately happened to the child.

For LMIC programs, sophisticated information technology should not become a prerequisite for progress. Simple, reliable systems that permit tracking of specimens, results, referrals, diagnoses, treatment, and outcomes may provide the foundation upon which more sophisticated systems can subsequently be developed.

**Screening Must Include the Child and Family:** Finally, De Costa et al. note that the ultimate measure of an NBS program should not be the number of specimens tested. Rather, it should be the number of newborns whose health and developmental potential have been improved.

Outcomes extend beyond survival. Newborns identified with disorders through screening may require rehabilitation, developmental services, assistive technologies, psychosocial support, surgeries, and long-term monitoring. Families will also need clear, timely, culturally appropriate, and non-stigmatizing communications. The WHO framework, therefore, appropriately places disability prevention, rehabilitation, developmental support, and family engagement within the broader screening pathway.

**From Framework to Implementation:** The WHO NBS framework represents a significant new milestone, but publication of a framework is only the beginning. The next challenge is implementation.

Each country should assess its readiness and identify conditions for which meaningful benefit can be delivered. Implementation includes establishing quality-assured screening and diagnostic pathways, developing sustainable financing, training the required workforce, creating effective referral systems, and establishing mechanisms for monitoring outcomes. Programs should also be able to expand progressively as capacity develops.

The objective should not be to reproduce the NBS program of a high-income country in an LMIC. Rather, countries should develop locally appropriate programs that meet internationally accepted principles of quality, equity, effectiveness, and continuity of care.

**Conclusions:** The various international NBS communities (bloodspot, hearing, cardiac, clubfoot, etc.) have an important role in this process. Professional organizations, reference laboratories, universities, development agencies, governments, and civil-society organizations can contribute expertise, education/training, proficiency testing, technology transfer, quality improvement, and program evaluation. But long-term success ultimately requires national ownership.

The WHA77.5 resolution provides the global mandate. The WHO framework provides an important roadmap. The responsibility now rests with Member States and the international NBS community to convert that roadmap into functioning programs.

The evolution from global policy to implementation represents an important advancement of NBS as a public health strategy. The success of NBS depends on the ability of a country to build integrated health systems that consistently translate early detection into improved outcomes for affected children. The WHO framework proposes integrated, scalable, and sustainable NBS services and identifies priorities that can determine whether comprehensive NBS will be fully realized in LMICs. The next era of NBS should not be defined simply by how many conditions can be detected. It should be defined by how effectively the identification, diagnosis, treatment, follow-up, and support for every newborn who needs help can be provided—regardless of where they are born.

## Abbreviations

The following abbreviations are used in this manuscript:

LMIC	Low- and middle-income country
NBS	Newborn screening (in the broadest sense—laboratory screening, physical examinations, or clinical tests)
WHA	World Health Assembly
WHO	World Health Organization
